# Lung ultrasound is a reliable method for evaluating extravascular lung water volume in rodents

**DOI:** 10.1186/s12871-015-0146-1

**Published:** 2015-11-12

**Authors:** Huan Ma, Daozheng Huang, Minzhou Zhang, Xin Huang, Shiyu Ma, Shuai Mao, Wenhui Li, Yanfen Chen, Liheng Guo

**Affiliations:** 1 The Department of Critical Care Medicine, Guangdong Provincial Hospital of Chinese Medicine, Guangzhou, Guangdong People’s Republic of China; 2 The Department of Critical Care Medicine, Guangdong Provincial Hospital, Guangzhou, Guangdong People’s Republic of China

**Keywords:** Acute lung injury, Oleic acid, Lung ultrasound, Extravascular lung water, Gravimetry, B lines

## Abstract

**Backgroud:**

Lung ultrasound (LUS) can diagnose extravacular lung water (EVLW) through the visualization of B lines in both humans and large animals. However, there are no published data on the use of ultrasound to detect EVLW in rats, the gold standard to evaluate of EVLW in rats is post-mortem gravimetric analysis. The present study was designed to determine the similarity between lung sonography and gravimetric measurements of EVLW in rats in an acute lung injury (ALI) model.

**Methods:**

Thirty male Sprague–Dawley rats were randomized into control and experimental groups. The B lines were measured byLUS at baseline. ALI was induced by the intravenous administration of oleic acid (OA) at a dose of 9 ul/100 mg, and controls were injected the same amount of isotonic saline. After 1 h, B-lines were measured by LUS in each rat following the induction of ALI. At the end of each experiment, both lungs were dissected, weighed and dried to determine wet/dry weight ratio according to the standard gravimetric methodology. Lung samples from three rats in each group were examined histologically.

**Results:**

B-lines were present in all rats from experimental group at 1 h point after OA injection. The statistical correlation between the two methods of assessing EVLW provided an *r* = 0.834 (*p* < 0.001). Repeatability studies of the LUS technique (Bland-Altman plots) showed good intra-observer and inter-observer reproducibility.

**Conclusion:**

The data suggest that, in an experimental rat model of ALI, B lines score as assessed by LUS can provide an easy, semi-quantitative, noninvasive.

Real-time index of EVLW which is strongly correlated to experimental gravimetric assessments.

## Backgroud

Assessment of extravascular lung water (EVLW) can be of great importance in critically ill patients as a sensitive indicator of acute lung injury (ALI) [1], acute respiratory distress syndrome (ARDS) [2], acute heart failure, and septic shock [3]. Therefore, reliable quantification of EVLW may provide valuable information to buide therapy in the intensive care setting [4]. Currently, there are several methods used to measure EVLW, which are generally limited by poor specificity, invasive techniques [5], and overall feasibility [6]. Transthoracic lung ultrasound (LUS), which is based on the visualization of multiple and diffuse B lines, is an established bedside test for assessing and grading EVLW in patients.

Despite the extensive application of LUS in human and large animals, there have been no previously published articles reporting the evaluation of EVLW by LUS in rats. As one of the most commonly used animals in experimental and clinical trials, rats are used in the study of a variety of lung-related illness, including septic lung injury, ALI and so on. Usually, pulmonary lung water is directly measured after sacrifice; however, it may be beneficial to measure EVLW over time or for the rats to survive for other experimental purposes. Potentially, LUS could be used to measure EVLW in vivo in a manner similar to cardiac ultrasound for small animals. Moreover, compared to large animals, like pigs [7] or bovines, the experimental use of rats is more economic.

Therefore, we hypothesized that LUS can be used to reliably measure EVLW in rats, as compared to the gold standard of evaluating EVLW by gravimetric assessment of the lungs post-mortem [8]. The present study was designed to investigate the level of agreement between lung sonography and gravimetric measurement of EVLW in rats with oleic acid-induced ALI.

## Methods

### Animal preparation

Thirty male Sprague–Dawley rats (aged 48 weeks, weighing between 225 g and 275 g; Animal Experiment Center, Southern Medical University) were obtained for this study. The study was performed in accordance with the approval and guidelines from the Institutional Animal Care and Use Committee of Guangdong Provincial Hospital of Chinese Medicine, Guangzhou University of Chinese Medicine. All rats were anesthetized with intraperitoneal pentobarbital sodium (50 mg/kg). A tracheotomy was performed, and a tracheal tube was inserted to a depth of 2 cm and secured in place to allow for mechanical ventilation using a rodent respirator (Harvard Apparatus, South Natick, MA). Rats were exposed to fractional inspired oxygen (FiO_2_) of 0.21 (room air), respiratory rate 73/min, tidal volume(TV) 4.5 ml/kg and 2 cm H_2_O positive end-expiratory pressure (PEEP). One of the carotid arteries was cannulated and connected to a physiological pressure transducer (Memscap AS, Cleveland, NOR) to record the systemic arterial pressure and heart rate on a physiological recorder (Power Lab, AD Instruments Pty Ltd, AU). The left internal jugular vein was catheterized for administration of intravenous fluid and/or drugs. Lines were fixed in place and heparinized saline (0.15 M, 2 U/ml) was infused (1 ml/h) to maintain the patency of the arterial and vein line. Continuous electrocardiographic monitoring was performed. Body temperature was continuously monitored via a rectal probe and maintained at 37 °C with a thermostatically controlled plate. Baseline data for cardiac function were collected using small animal ultrasound, which is an animal-dedicated machine (VisualSonics Vevo® 2100 System; VisualSonics Inc, Toronto, Ontario, Canada).

### Hemodynamic variables

Arterial blood pressure and heart rate were recorded with reference to atmospheric pressure at the mid-thoracic level at end expiration. All variables were displayed and collected on a personal computer.

### Study design

Animals were randomly separated into either a control group (*n* = 15) or an ALI group (*n* = 15). ALI was induced by intravenous injection of oleic acid (OA) (Sigma-Aldrich, United Kingdom), which was prepared as a 1:1 mixture with pure ethanol [9]. The rats were placed in the supine position and infused via the left internal jugular vein with OA (9 ul/100 g) over 5 min. The control group was infused with an equal volume of 0.9 % normal saline (NS). Additional pentobarbital sodium (25 mg/kg) was administered every 45 min, providing a constant depth of anesthesia as monitored by the animal’s cardiorespiratory and reflex responses. Cardiac function was evaluated by echocardiography, and B lines were assessed by LUS before the experiment and at 1 h following the injection of OA/NS. Repeatability studies of the LUS technique were also conducted between the intra-observer and the inter-observer. The animals were then euthanized by exsanguination under anesthesia. Lungs were removed immediately post-mortem for further analysis. To confirm the presence of injury, lung tissue was collected for histologic analysis for three rats from each group.

### Chest sonography and image analysis

**Fig. 1:**
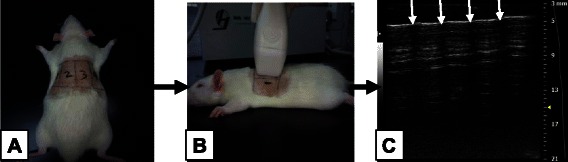
**a** LUS image obtained from the rats’ dorsal wall which was divided into four zones. **b** Lung scanning was performed in the vertical plane, from zone one to zone four. Four times scanning can cover the whole rat lung. **c** Five to six intercostal space were present in each scan. In the ultrasound image, four arrows represent rib shadow, the white line between arrow designates the pleural line

LUS images were obtained using the small animal ultrasound, an animal-dedicated machine (VisualSonics Vevo® 2100 System; VisualSonics Inc, Toronto, Ontario, Canada) with the cardiac probe (30 MHz). In contrast to some other models, the rats were evaluated while in the prone position. Images were obtained from the dorsal wall in prone position (Fig. 1a). The reason for such a position was that heart is close to the anterior wall, and the beating heart may interfere with the quality of LUS images.

In humans, the 28-rib space technique is used to semi-quantitatively evaluate the volume EVLW [10], and the number of B-lines are counted at four sites (parasternal, mid-clavicular, anterior-axillary and mid-axillary lines) in each space from the second to the fourth intercostal spaces of the left hemithorax and the second to the fifth intercostal spaces on the right (3*4 + 4*4 = 28). This evaluation is performed at precisely defined lines for accurate measurements. In rats, the “28-rib space” technique was modified due to the smaller area of the rats’ backside. The LUS consisted of bilateral scanning of the posterior(dorsal)and lateral chest walls. Each hemithorax was divided into two zones: (1) from the posterior axillary line to the scapular line and (2) from the scapular line to the paravertebral line. A total of four scans were performed for each rat. Lung scanning was performed in the vertical plane on the rat’s dorsal wall (Fig. 1b). In each scan, five to six intercostal spaces were present on the screen. The probe was oriented in the “up and down” position to allow for whole lung imaging, which consistently demonstrated seven intercostal spaces (Fig. 1c). In summary, the lung was conceptually divided into four parts, and each parts contained seven intercostal spaces. This manner was analogous to the “28-rib space” evaluated in humans, with the only difference being that the vertical scanning plane was used in rats while the horizontal scanning plane is typically used in humans. The scanning sites were marked by a drawing pen in order to put the probe at exactly the same anatomical point each time.

### B lines scoring

**Fig. 2:**
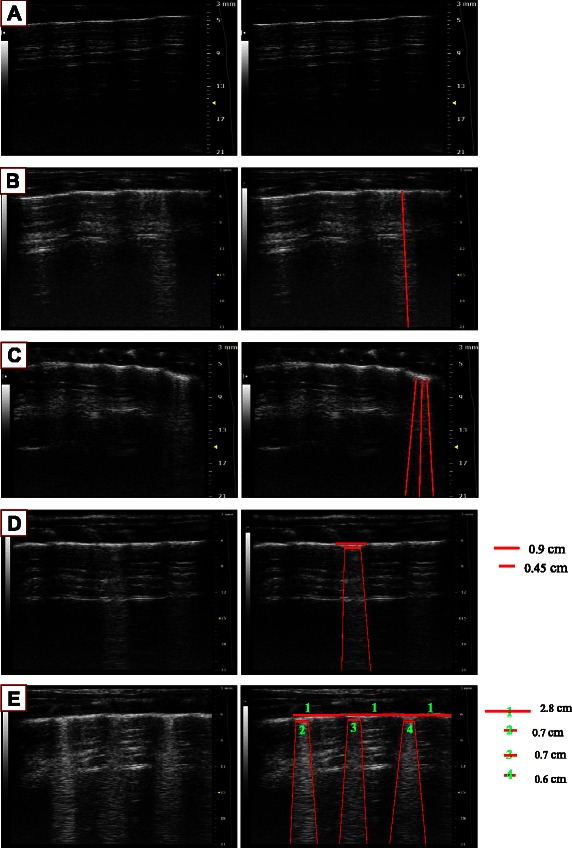
How to calculate the B line score **a** 0 B line (Score = 0). **b** 1 B line (Score = 1). **c** 3 B lines (Score = 3). **d** Confluent B lines assesse the percentage of the rib space and divided it by ten. Score = 045 cm/0.9 cm × 10 = 5. **e** Confluent B lines present in more than one rib spaces: Add each score together to get the whole score in one scan. Score = 0.7 cm/2.8 cm × 10 + 0.7 cm/2.8 cm × 10 + 0.6 cm/2.8 cm × 10 = 10.15 ≈ 10. The whole score of the rat lung semiquantified by LUS is the sum of four scores in each scan

For each scan, the A lines or B lines was recorded at baseline [11], and 1 h after the injection of OA or NS. The A lines were present as repetitive horizontal artifacts parallel to the pleural line and arising from it, caused by the preponderance of air over liquid in the lung parenchyma. The presence of A lines was given a score of zero, indicating a normal LUS pattern. The B lines defined as discrete laser-like vertical hyperechoic reverberation artifacts that arise from the pleural line (previously described as “comet tails”), extending to the bottom of the screen without fading, and move synchronously with lung sliding [10]. The B lines score was evaluated as follows: at each intercostal space, the number of B lines was counted (ranging from zero to ten, corresponding to a confluency of signal) and then the percentage of the rib space occupied by B-lines was determined and divided by ten (see Fig. 2). The B lines scores was evaluated by two physicians trained in the evaluation of chest ultrasound, and a consensus was reached when there was disagreement.

### Experimental gravimetric measurements of lung water volume

Blood samples were collected from abdominal aorta immediately after sacrifice and subjected to centrifugation (3000xG for 10 min). The resulting serum was frozen at−20 °C until biochemical analysis was performed. Biochemical markers (including hemoglobin concentration, haematocrit, and the wet/dry weight ratio of the blood) were measured in the serum by an automated biochemical analyzer (sysmex xs-800i, STAC Medical Science & Technology Co, Ltd,Japan). The lungs were removed immediately post-mortem, drained of blood and weighed. An equal amount of water was added to induce hemolysis. The lungs and the added water were homogenized using a commercial blender and an electric homogenizer. The dry weights of the blood, homogenate and supernatant were determined after drying at 60 °C for 72 h. After that, the fractional water contents were calculated and used to calculate the total lung water and the intravascular lung water. EVLW was defined as the difference between the total lung water and the intravascular lung water [12].

### Histopathological examination

In each group, lung tissue of three rats as immersed in 10 % formaldehyde fixative for 24 h and then washed with water. For light microscopic examination, lung tissue was dehydrated with graded alcohol and then embedded in paraffin at 60 °C. Histologic sections were then stained with hematoxylin and eosin.

### Reproducibility studies

The inter-observer and intra-observer reproducibility of B lines score was assessed in all the ALI group. Both physicians are expert echocardiography technicians, one of them had received specific training on chest LUS, and the second one was trained by the first one (in a 2 h practical session) to measure the B lines score. The reliability of B lines score over time was investigated in a a series of 15 rats after LPS injection.

### Statistical analysis

SPSS v13.0 software was used for data analysis. All data were presented as mean ± SD. Differences between the control and the OA group were tested using Student’s *t*-test. Correlation between the B lines score and gravimetric measurement values was determined using Pearson correlation coefficient analysis. The agreement between B lines score assessed in intra-observers and inter-observers was tested by the Bland-Altman method. For all the statistical analyses, significance was determined at the 0.05 level.

## Results

### Hemodynamic and respiratory effects

Hemodynamic and respiratory variables are shown in Table 1 for the two groups of animals, treated with NS or OA and euthanized after 1 h (Table 1).

**Table 1:** Hemodynamic, gravimetry and lung ultrasound variable at two groups

	Control	OA
Heart rate (bpm/min)	427 ± 17	389 ± 84
Systemic blood pressure(mmHg)	114 ± 11	67 ± 9*
EVLWI	2.02 ± 0.22	3.92 ± 0.68*
B-lines score	1.58 ± 0.79	15.75 ± 4.90*

* = *p* < 0.01

### B lines score obtained from LUS

**Fig. 3:**
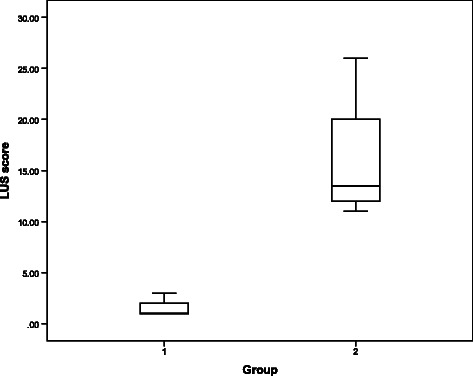
LUS findings between the two groups

Each assessment of B-lines was performed in less than 5 min, with full feasibility of 100 %. At baseline, the mean number of B-lines in the control group was 1.6 ± 0.8, after OA injection, and the number of B-lines increased bilaterally over time and more markedly at 1 h later with a mean of 15.8 ± 4.9 (Fig. 3).

### Gravimetric measurements

**Fig. 4:**
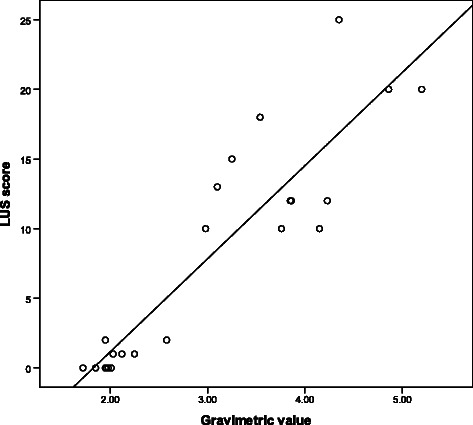
The correlation between gravimetric values (*x-axis*) and LUS findings (*y-axis*) in the 24 rats

The extravascular fluid content of the lung samples expressed by the wet-dry ratio significantly increased after the OA injection compared with the sham-operated control group (X vs. Y, *p* < 0.05). The correlation between the two methods of assessing EVLW is shown in Fig. 4 and provides an *r* = 0.834 (*p* < 0.001).

### Pathological findings

**Fig. 5:**
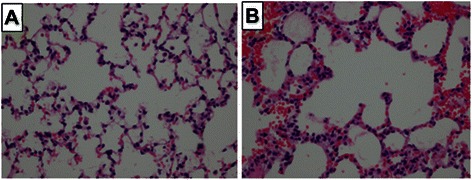
The pathology of rat lung 1 h after the onset of intravenous infusion of saline (**a**) or 9 ul/100 mg OA (**b**). Note the presence of hemorrhage and inflammatory infiltrates in the lungs of the rat treated with oleic acid

The histopathological changes between the NS (control) group and experimental group are shown in Fig. 5. Compared with the NS group, the experimental group demonstrated severe pulmonary hemorrhagic edema with inflammatory cell infiltration (Fig. 5).

### Reproducibility studies

**Fig. 6:**
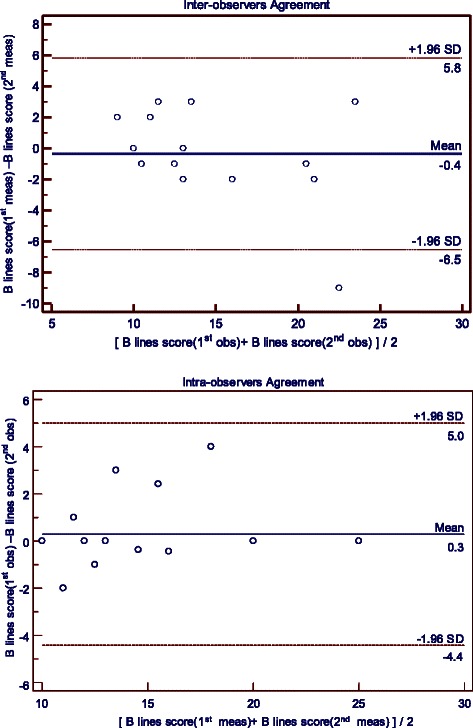
Reproducibility Studies of B lines scores. Reproducibility studies of the B lines scores over time (upper graph) in 15 rats. Inter-observer agreements were ascertained in a series of 15 independent measuments (bottom graphs). All graphs are Bland-Altman plots

The reproducibility of the B lines score over time in 15 rats was good in that the difference between the 2 measurements was always less than 2 SD of the average measurement (concordance index = 0.86, 95 % confidence interval: 0.62 to 0.96) (Fig. 6). The agreement between the expert and the naive observer was quite satisfactory: in over 15 independent measurements, only in 1 rats did the between-observer difference exceed 2SD (Fig. 6).

## Discussion

The major findings of this study are that B-lines can be reliably detected in a rat model of ALI, although both the position of rat and the scanning manner are different from large animals. The B line score (The “28-scanning-site” method) assessed by LUS provides a simple, semiquantitative, and noninvasive index of lung water accumulation which significantly correlates with experimental gravimetric assessment.

Various studies have focused on the correlation between ultrasound and other technique to measure elevated EVLW. These techniques include radiographic and quantitative computed tomography [13], as well as invasive methods of lung water estimation [14–16] and hydrostatic pressure measurements [16, 17]. Additionally, assessment of EVLW using ultrasound have been completed and shown to be reliable in other animal models, including pigs [7] and bovines [18]. However, to the best of our knowledge, no published articles have yet tested the accuracy of LUS in rats with ALI/ARDS, and this is the first report to assess the validity of LUS in diagnosing rats with ALI/ARDS.

Sonography is a surface-imaging technique, and LUS examination is limited to the lung periphery. This method of observation is currently used in critically ill and emergency situations and is useful to characterize lesions which extend to the lung surface. In humans, this phenomenon is well-explained [19]. B lines correspond to the ultrasound signs of loss of aeration and increase in density of the lung periphery. Intuitively, the same limitations for humans may extend to pigs, rats and other animals because B lines are artifacts that are generated from the interaction between the ultrasound beam and the lung periphery.

The question remains as to how much of the peripheral lung is representative of systemic lung injury. In pulmonary edema the lung periphery is highly and homogeneously involved. This explains why measuring B lines, which are signs of loss of aeration of the lung periphery, is useful to semiquantify EVLW and the loss of aerations of the whole lung. In other conditions where the distribution of the lung involvement is not homogeneous or spares the lung periphery, B lines will be less correlated with the severity of the condition. This limitation does not change depending on the dimension or the thickness of the lungs, and, as our study shows, remains even in rats. During the course of our research, we found that the methods of LUS and gravimetric measurement to assess EVLW are entirely consistent with one another, verifying that LUS can be used in ALI/ARDS rats despite of the same limitations seen in human measurements.

According to “International evidence-based recommendations for point-of-care lung ultrasound”, the term “B-pattern” should be used (rather than “lung rockets” or “B-PLUS”) in the description of multiple B-lines in patients with interstitial syndrome. For a more precise quantification of interstitial syndrome, the 28-scanning-site technique can be useful, especially in cardiology and nephrology settings. We also performed the evaluation of intercostal spaces since it is also a standardized measurement in humans. The only difference is the width of the spaces that in rats are far inferior than in bigger animals [7, 20] and in humans. Thus, there is no difference for the purpose of the analysis of lung surface between ribs.

## Conclusions

Our data confirm that LUS can be used in rats to detect EVLW, and provide support for the use of LUS to provide a simple, semiquantitative, and noninvasive index of lung water accumulation, which was correlates with experimental gravimetric assessment in rats. These findings may reduce the number of animals needed for ARDS experiments and make future longitudinal studies possible.

## Abbreviations

LUS: Lung ultrasound
EVLW: Extravacular lung water
ALI: Acute lung injury
ARDS: Acute respiratory distress syndrome
FiO_2_: Fractional inspired oxygen
TV: Tidal volume
PEEP: Positive end-expiratory pressure
NS: Normal saline
